# Research on the consumption of sugar-sweetened beverages among seventh grade students in Beijing based on the social ecological model

**DOI:** 10.3389/fnut.2026.1817462

**Published:** 2026-06-23

**Authors:** Jiarui Zheng, Lulu Meng, Yiran Li, Yan Zhang, Ruoxiang Cao, Jiali Duan, Liyu Huang

**Affiliations:** 1 School of Public Health, Capital Medical University, Beijing, China; 2 Office of Research and Teaching Administration, Beijing Center for Disease Prevention and Control, Beijing, China; 3 School of Public Health, Hebei Medical University, Shijiazhuang, China; 4 Institute of Nutrition and Food Hygiene, Fengtai District Center for Disease Prevention and Control, Beijing, China; 5 Institute of Nutrition and Food Hygiene, Beijing Center for Disease Prevention and Control, Beijing, China

**Keywords:** influencing factors, social ecological model, students, sugar-sweetened beverages, teenagers

## Abstract

**Background:**

Teenagers are in a crucial stage of growth and development. The intake of sugar-sweetened beverages (SSB) will lead to various health problems such as obesity, dental caries and diabetes, seriously affecting their physical health.

**Objectives:**

This study aims to investigate the consumption of SSB among seventh grade students in Beijing. Utilizing the social ecological model, this study examines the factors influencing consumption at four levels: individual, interpersonal, environmental, and policy.

**Methods:**

This cross-sectional study employed a multi-stage stratified cluster sampling method. SSB consumption was defined as consuming sugar-sweetened beverages at least once per week. A combination of questionnaires and semi-structured interviews, based on the social ecological model, was used to investigate the characteristics of SSB consumption and its multi-level influencing factors. Chi-square tests and logistic regression analyses were used to analysis.

**Results:**

A total of 1,212 participants were included in the analysis. The prevalence of SSB consumption among seventh grade students in Beijing was 90.6%, with 61.5% consuming these beverages once or more daily. Logistic regression analysis revealed that knowledge of SSB (*OR* = 1.881, 95% *CI* [1.082–3.270]), peer influence on purchasing (*OR* = 1.803, 95% *CI* [1.167–2.785]), sharing beverages among peers (*OR* = 1.841, 95% *CI* [1.199–2.826]), parental support for SSB consumption (*OR* = 1.629, 95% *CI* [1.072–2.476]) and parental consumption of SSB (*OR* = 3.998, 95% *CI* [2.500–6.395]) were associated with increased SSB consumption among students. Interviews with teachers indicated that the majority do not support SSB consumption among students.

**Conclusion:**

The prevalence of SSB consumption among seventh grade students in Beijing is high. The consumption of SSB is closely related to students’ cognition, parents’ attitudes and behaviors, peer behaviors, and health promotion policies. Future initiatives should prioritize enhancing health education to improve students’ nutritional literacy and promoting policies to reduce SSB consumption.

## 1 Introduction

Sugar-sweetened beverages (SSB) refer to drinks that have added sugars during their production process, with a sugar content of more than 5% (1), such as carbonated drinks, sugar-sweetened juices, and sugar-sweetened dairy beverages. Added sugars are defined as sugars that are artificially added to foods, characterized by their sweetness, including monosaccharides and disaccharides. The “Dietary Guidelines for Chinese Residents (2022)” (1) recommend that healthy adults limit their intake of added sugars to no more than 50 g per day, with added sugars providing no more than 10% of total daily energy requirements. In both adults and children, WHO strongly recommends reducing the intake of free sugars to less than 10% of total energy intake (2).

With economic development and rising living standards, the global consumption of SSB has shown a continuous increase, especially among children and adolescents. Studies showed that in 2018, the average weekly intake of SSB globally was 892.8 g (3). Between 199 and 2018, the intake of SSB among children and adolescents aged 3 to 19 increased by 23% (3). Currently, the consumption rate of SSB among urban adolescents aged 12 to 17 in China is 68.3% (4), with an average daily intake of 189.91 g (1), significantly higher than the global average. In Beijing, the proportion of primary and secondary school students consuming SSB at least once a week ranged from 43.6 to 67.3%. More than half of middle school students consume SSB more than twice a week, and nearly 5% consume daily (5).

For adolescents in their developmental stages, excessive consumption of SSB poses significant risks. Long-term excessive sugar intake can increase the risk of overweight and obesity among adolescents (6). The sugars in SSB can lead to oral health issues, such as cavities and periodontal disease (7). Maintaining a high-sugar diet over the long term may affect the normal functioning of the pancreas, increasing the risk of diabetes, especially among genetically predisposed adolescents (8). Moreover, excessive intake of SSB is associated with an increased risk of various diseases, including cardiovascular diseases (9), hypertension (10, 11), and chronic kidney disease (12). These conditions not only impact individual quality of life but also impose a significant healthcare burden on society (13).

Currently, large number of studies have explored the influencing factors of consumption. A study (14) based on the Theory of Planned Behavior indicates that the consumption of SSB is most closely related to personal behavioral intentions, followed by attitude, perceived behavioral control, and subjective norms. Research by Yuhas et al. (15) on SSB consumption among American adolescents showed that the accessibility of SSB at home was closely related to their intake. A systematic review by Mazarello et al. (16), based on the social ecological model, indicated that adolescent consumption of SSB was related to unhealthy lifestyle habits, parental age, income, household accessibility, school accessibility, and the availability of SSB in the surrounding environment. A survey (17) involving 6,015 adolescents aged 6 to 17 in China showed that the intake of SSB among adolescents was significantly related to the economic development level of their cities, family income, individual dietary habits, and parents’ consumption attitudes. Consumption levels in urban areas were generally higher than in rural areas, and high-income families consumed more than low-income families, while individuals with poor dietary habits tended to consume more SSBs (17). A cohort study (18) involving 904 students indicated that parents’ consumption habits of SSBs were significantly related to students’ consumption, with boys being more influenced than girls.

**Figure 1:**
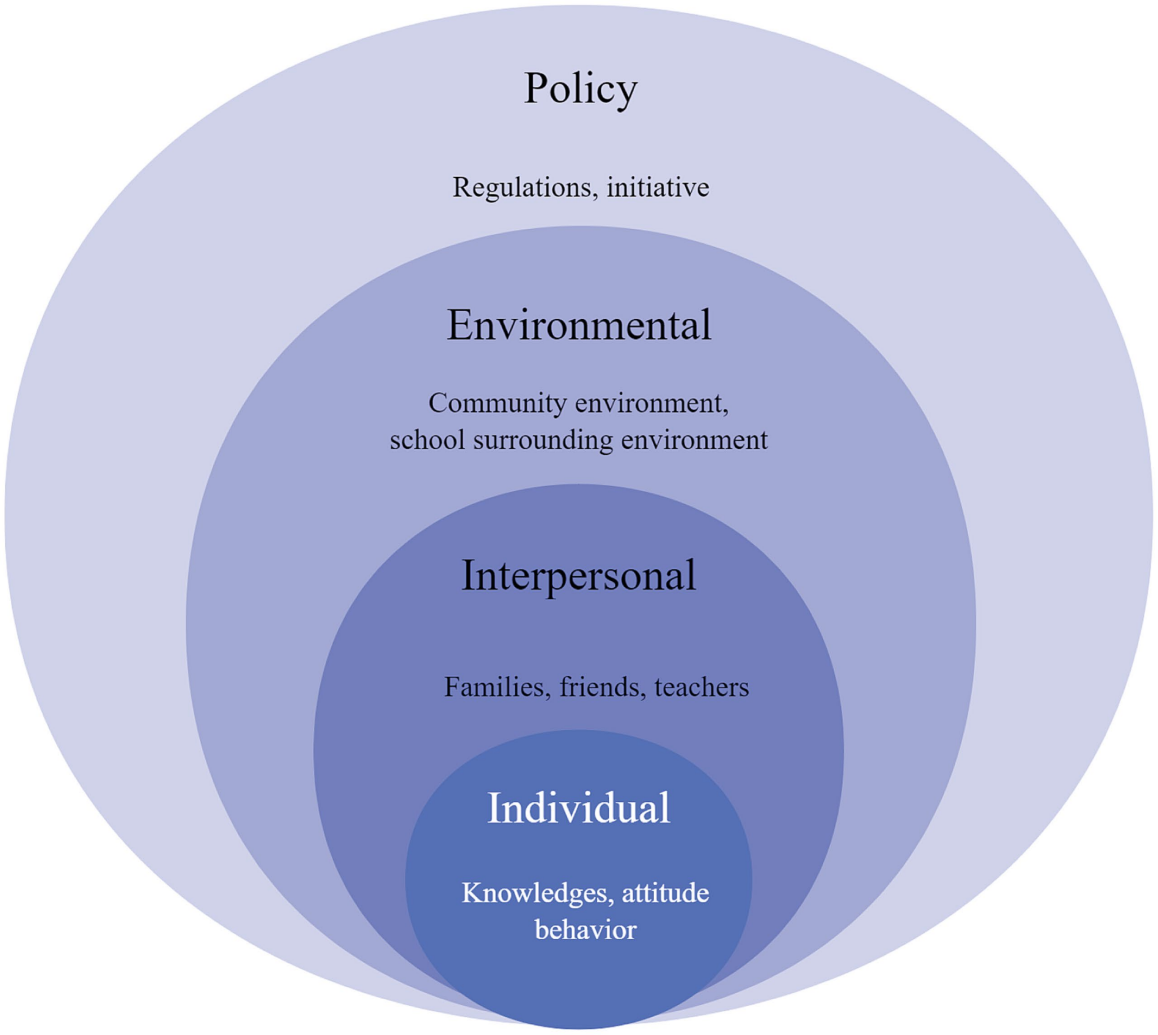
The social ecological model.

The Social Ecological Model (SEM) serves as a comprehensive theoretical framework that can be used to analyze and understand the complex interactions between individual behavior and environmental and social policy factors. This model emphasizes that individual behavior is influenced by the interaction of multiple levels of factors, involving individual, interpersonal, environmental, and social policy aspects (19). Such a multidimensional analytical framework can comprehensively capture various influencing factors, revealing direct effects, indirect effects, and mediating effects between factors, thus providing an in-depth analysis of the complex relationships behind behaviors (Figure 1).

Currently, research in China primarily focuses on the individual level, with few articles addressing the impact of external environmental factors on consumption. This study utilizes surveys of seventh grade students in Beijing regarding their consumption of SSB, clarifying the status and related influencing factors of SSB consumption among these students from individual, interpersonal, environmental, and policy perspectives. It aims to explore the mechanisms of influence among these factors and lays the foundation for designing a series of practical health intervention strategies and measures.

## 2 Materials and methods

### 2.1 Study design and participants

This study used a cross-sectional design and was conducted in Beijing, China, between October 1, 2024 and November 30, 2024. A multi-stage stratified cluster sampling approach was applied. Based on administrative divisions and geographic characteristics, Beijing was categorized into three regions: central urban, remote urban, and suburban areas. Two junior middle schools were selected from each region.

All seventh-grade students (aged 11–14 years), their primary caregivers, and teachers in the selected schools were invited to participate. Inclusion criteria were: (1) seventh-grade students and their caregivers residing in Beijing; (2) ability to complete the questionnaire independently; (3) provision of written informed consent. Students who were on leave or declined participation were excluded.

Based on literature and the Report on Nutrition and Health Status Monitoring of Chinese Residents (2015–2017) (4), which indicates a SSB consumption rate of 68.3% among urban children and adolescents aged 12 to 17 in China, we set *p* = 68.3%, *Z*/2 = 1.96, *d* = 0.1p. The design effect for cluster sampling was deff = 2. Using Equation 1, considering an estimated invalid questionnaire and non-response rate of 10%. We considered factors such as population, economy, geography, and educational resources, Beijing’s administrative districts were categorized into three levels: central urban, remote urban, and suburban areas, resulting in a total sample size of 1,182 individuals across the three regions.


$$n=\frac{Z_{\alpha /2}^{2}\times p(1-p)}{d^{2}}\times \text{deff}$$ (1)

A total of 1,307 student questionnaires and 1,307 caregiver questionnaires were distributed. After data cleaning, 1,212 valid matched student–parent pairs were included in the final analysis, yielding a response rate of 92.73%. In addition, 49 teachers participated in qualitative interviews. This study was approved by the Ethics Committee of the Beijing Center for Disease Control and Prevention (BJCDC2024031). Written informed consent was obtained from all participants and from the guardians of minor students.

### 2.2 Quantitative data collection

#### 2.2.1 Questionnaire development and validation

The student questionnaire on SSB consumption and the caregiver questionnaire on the family environment were developed based on the Social Ecological Model, and were further refined through expert consultation. A pilot survey was conducted before the main study, with a sample size of 150 participants.

The reliability and validity of the questionnaires were assessed through a pilot study. Internal consistency and construct validity were evaluated separately for the student and caregiver questionnaires. For the student questionnaire, the 17-item attitude scale demonstrated well internal consistency, with a Cronbach’s *α* of 0.819. The KMO value was 0.824, and Bartlett’s test of sphericity was statistically significant (*p* < 0.001). For the caregiver questionnaire, the 12-item attitude scale showed acceptable internal consistency (Cronbach’s α = 0.715). The KMO value was 0.772, and Bartlett’s test was also statistically significant (*p* < 0.001). These results indicate that both questionnaires demonstrated satisfactory reliability and validity and were suitable for use in this study.

#### 2.2.2 Definition of sugar-sweetened beverages

SSBs were defined as beverages containing added sugars during processing, including both pre-packaged beverages and freshly prepared beverages. Pre-packaged beverages included carbonated drinks, sweetened fruit and vegetable juices, carbonated beverage, tea drinks, dairy beverage, plant-protein drink, special use beverage, coffee and other pre-packaged drinks, while freshly prepared beverages included coffee, fruit tea, pure tea, milk tea, fruit juice, specialty tea, yogurt/milk, carbonated drink and other freshly prepared drinks. Pre-packaged beverages were classified based on product labeling, and freshly prepared beverages were classified as SSBs only if participants reported that sugar was added during preparation. Beverages identified as sugar-free or artificially sweetened were excluded.

#### 2.2.3 Outcome and independent variables

The primary outcome was SSB consumption in the past week, defined as consuming SSB at least once per week in the past week (20). Participants were asked to report the frequency of SSB consumption over the past week using predefined categories, including “times per day,” “times per week,” and “rarely or never,” which were subsequently standardized to times per week. To further examine potentially risky consumption patterns, a secondary outcome variable was constructed based on consumption frequency. High-frequency SSB consumption was defined as consuming SSBs ≥ 7 times per week. Lower-frequency SSB consumption was defined as consuming SSBs < 7 times per week. In addition, participants reported the average amount consumed per occasion (in milliliters), which was used to estimate average daily intake. However, given the potential bias associated with self-reported consumption, particularly for intake volume, consumption frequency was considered a more reliable measure and was therefore used as the basis for defining the high-frequency consumption outcome.

Independent variables were assessed across multiple levels. At the individual level, data were obtained from student questionnaires and included demographic characteristics, SSB-related knowledge (10 items), attitudes (17 items measured using a Likert scale for 4 domains), and health-related behaviors. At the interpersonal level, peer-related variables (peer consumption, peer-influenced purchasing, and sharing behaviors) were reported by students, while parental variables (knowledge, attitudes, and SSB consumption) were collected through matched caregiver questionnaires. At the environmental level, variables were assessed through student reports and field observation, including the availability of SSB within 500 meters of the residence and the presence of SSB sales points within 100 meters of schools. At the policy level, data included exposure to health warning labels and their perceived influence on beverage purchasing. Details of questionnaire items and scoring methods (21) are provided in Supplementary Table S1.

### 2.3 Qualitative data collection and analysis

Semi-structured interviews, including in-depth individual interviews and focus group discussions, were conducted with 49 teachers using purposive sampling. The interview guide (Additional File 1) covered perceptions of SSB-related health risks, attitudes toward student consumption, and suggestions for intervention. All interviews were conducted in a private setting and lasted approximately 25–30 min.

All interviews were audio-recorded and transcribed verbatim. Transcripts were imported into NVivo 14.0 for data management and coding. Data were analyzed using grounded theory. Open, axial, and selective coding were used to develop and refine thematic categories. Two researchers independently coded the transcripts, and discrepancies were resolved through discussion to ensure consistency.

Qualitative data were incorporated within the social ecological model framework to complement the quantitative analysis, particularly by providing additional insights into interpersonal and organizational-level factors.

### 2.4 Statistical analysis

Quantitative data were analyzed using SPSS 26.0. Descriptive statistics were used to summarize participant characteristics, SSB consumption patterns, consumption frequency, and average daily intake. Chi-square tests were used to examine associations between SSB consumption and categorical variables. For continuous variables, the Kruskal–Wallis *H* test was used to compare differences in average daily SSB intake across regions.

A binary logistic regression model was used to identify factors associated with SSB consumption in the past week. The dependent variable was defined as whether the student had consumed SSBs in the previous week (1 = yes, 0 = no). Candidate predictors were selected based on the social-ecological framework and included individual-level, interpersonal-level, and policy-level factors. Individual-level factors included knowledge of SSBs and health awareness. Interpersonal-level factors included peer SSB consumption, purchasing SSBs because of peers’ purchases, beverage sharing, parents’ attitudes toward students’ SSB consumption, and parents’ SSB consumption. Policy-level factors included the perceived impact of point-of-sale health warning labels on beverage purchases and discouraging family members or friends from drinking SSBs after seeing health warning labels. Gender, region, only-child status, primary caregiver, and caregiver education level were entered as demographic covariates in the forward stepwise model-selection procedure. The logistic regression model was specified as follows Equation 2:


$$\text{logit}(P)=\ln(\frac{P}{1-P})=\beta_{0}+\beta_{1}X_{1}+\beta_{2}X_{2}+\ldots +\beta_{n}X_{n}$$ (2)

A secondary analysis was conducted using high-frequency SSB consumption as the outcome, defined as ≥7 times/week. Binary logistic regression was performed using the same independent variables and modeling procedures as described above.

Model fit was assessed using the Hosmer–Lemeshow goodness-of-fit test. Multicollinearity among independent variables was evaluated using variance inflation factors (VIF). All statistical tests were two-sided, and a *p* value < 0.05 was considered statistically significant.

## 3 Results

### 3.1 General information

A total of 1,212 valid matched student–parent pairs were included in the final quantitative analysis. Among the respondents, there were 610 boys (50.3%) and 602 girls (49.7%); 402 students from urban central areas (33.2%), 412 from remote areas (33.9%), and 398 from suburban areas (32.9%); most of students held urban household registration (91.8%); and 714 were the only children (58.9%). The main caregivers of seventh grade students in Beijing are primarily parents, comprising 98.3%, with 61.8% of these caregivers having an education level of bachelor’s degree or above (Table 1).

**Table 1:** Demographic characteristics of seventh grade students in Beijing [*n* (%)].

Demographic characteristics	Total	Region		
		Central urban	Remote urban	Suburban
Gender				
Male	610(50.3)	216(53.7)	202(49.0)	192(48.2)
Female	602(49.7)	186(46.3)	210(51.0)	206(51.8)
Household registration type				
Urban	1,113(91.8)	395(98.3)	365(88.6)	353(88.7)
Rural	99(8.2)	7(1.7)	47(11.4)	45(11.3)
Only child				
Yes	714(58.9)	256(63.7)	255(61.9)	203(51.0)
No	498(41.1)	146(36.3)	157(38.1)	195(49.0)
Primary caregiver				
Parents	1,192(98.3)	389(96.8)	410(99.5)	393(98.7)
Grandparents or others	20(1.7)	13(3.2)	2(0.5)	5(1.3)
Educational level of primary caregiver				
High school and below	167(13.8)	27(6.7)	89(21.6)	51(12.8)
College/technical college	296(24.4)	60(14.0)	126(30.6)	110(27.6)
Bachelor’s degree and above	749(61.8)	315(78.4)	197(47.8)	237(59.5)
Total	1,212(100)	402(33.2)	412(34.0)	398(32.8)

In addition, 49 teachers from central urban, remote urban and suburban areas participated in qualitative interviews. Participants included school leaders, health teachers, homeroom teachers, and subject teachers.

### 3.2 Consumption of sugar-sweetened beverages among seventh grade students in Beijing

#### 3.2.1 Prevalence of sugar-sweetened beverages consumption

The prevalence of SSB consumption among seventh grade students in Beijing is 90.6%. Among them, both students from urban central areas and remote urban areas have a higher prevalence of SSB consumption compared to suburban students (92.3% vs. 92.3% vs. 87.2%, *p* < 0.05) (Table 2).

**Table 2:** The consumption of sugar-sweetened beverages [*n* (%)].

Demographic characteristics	Survey sample (*n* = 1,212)	Consumption of Sugar-sweetened beverages		*χ* ^2^	*P*-value
		Yes	No		
Gender				0.073	0.787
Male	610(50.3)	554(90.9)	56(9.1)		
Female	602(49.7)	544(90.4)	58(9.6)		
Region				8.079	0.018
Central urban	402(33.1)	371^a^(92.3)	31^a^(7.7)		
Remote urban	412(34.0)	380^a^(92.3)	32^a^(7.7)		
Suburban	398(32.9)	347^b^(87.2)	51^a^(12.8)		
Only Child				0.001	0.975
Yes	714(58.9)	647(90.6)	67(9.3)		
No	498(41.1)	451(90.6)	47(9.4)		
Primary Caregiver				0.463	0.496
Parents	1,192(98.3)	1,079(90.6)	113(9.4)		
Grandparents or Others	20(1.7)	19(95.0)	1(5.0)		
Educational Level of Primary Caregiver				3.764	0.152
High School and Below	167(13.8)	151(90.4)	16(9.6)		
College/Technical College	296(24.4)	260(13.8)	36(12.1)		
Bachelor’s Degree and Above	749(62.0)	687(91.7)	62(8.3)		
Total	1,212(100)	1,098(90.6)	114(9.4)		

a, b indicated subsets of each variable after LSD pairwise comparison; different letters indicate statistically significant differences.

The prevalence of consumption for pre-packaged beverages among seventh grade students in Beijing is 84.4%. The top three types of beverages ranked by prevalence from highest to lowest are tea drinks (48.4%), 100% fruit and vegetable juice (47.9%), and carbonated drinks (43.0%) (Supplementary Table S2). The prevalence of consumption for freshly made beverages is 79.6%, with the top three types ranked from highest to lowest being fruit tea beverages (42.0%), pure tea beverages (41.1%), and milk tea beverages (39.2%) (Supplementary Table S2).

#### 3.2.2 Distribution of sugar-sweetened beverages consumption frequency

Regarding total SSB consumption, the distribution of consumption frequency showed that 61.5% of students consumed SSB at least once per day, 13.1% consumed 4–6 times per week, 16.0% consumed 1–3 times per week, and 9.4% consumed less than once per week. The percentage of students from remote areas consuming SSB daily is higher than that of suburban students (64.5% vs. 56.0%, *p* < 0.05).

In terms of freshly made SSB, the distribution of consumption frequency showed that 34.5% of students consumed them at least once per day, 16.5% consumed 4–6 times per week, 28.6% consumed 1–3 times per week, and 20.4% consumed less than once per week. Students from remote areas consume them 4–6 times per week at a higher rate than suburban students (20.4% vs. 13.3%, *p* < 0.05) (Table 3).

**Table 3:** Distribution of the frequency of sugar-sweetened beverages consumption [*n* (%)].

Consumption frequency	Total	Region			*χ* ^2^	*P*-value
		Central urban	Remote urban	Suburban		
Sugar-sweetened beverages					14.330	0.026
<1 times/week	114(9.4)	31(7.7)^a^	32(7.7)^a^	51(12.8)^a^		
1 ~ 3 times/week	194(16.0)	60(14.9)^a^	58(14.0)^a^	76(19.1)^a^		
4 ~ 6 times/week	159(13.1)	54(13.5)^a^	57(13.8)a	48(12.1)^a^		
≥1 times/day	745(61.5)	257(63.9)^a,b^	265(64.5)^b^	223(56.0)^a^		
Pre-packaged beverages					11.617	0.071
<1 times/week	190(15.6)	64(15.9)	52(12.6)	74(18.5)		
1 ~ 3 times/week	334(27.5)	106(26.4)	115(27.9)	113(28.4)		
4 ~ 6 times/week	189(15.7)	74(18.4)	56(13.6)	59(14.8)		
≥1 times/day	499(41.2)	158(39.3)	189(45.9)	152(38.3)		
Freshly made beverages					20.938	0.002
<1 times/week	248(20.4)	64(15.9)^a^	79(19.1)^a^	105(26.3)^b^		
1 ~ 3 times/week	344(28.6)	120(30.0)^a^	110(26.5)^a^	114(28.6)^a^		
4 ~ 6 times/week	200(16.5)	82(20.4)^a^	65(16.0)^a,b^	53(13.3)^b^		
≥1 times/day	420(34.5)	136(33.7)^a^	158(38.4)^a^	126(31.8)^a^		

a, b indicated subsets of each variable after LSD pairwise comparison; different letters indicate statistically significant differences.

#### 3.2.3 Average daily consumption of sugar-sweetened beverages

Analysis of students who consumed SSB at least once in the past week shows that the average daily consumption of total SSB among seventh grade students in Beijing is [392.86(178.57, 809.28)] mL/d. The average daily consumption of pre-packaged beverages is [201.99(85.71, 427.55)] mL/d, and the average daily consumption of freshly made beverages is [157.14(57.14, 350.00)] mL/d. The average daily consumption of total SSB (*H* = 17.514, *P* < 0.001), pre-packaged beverages (*H* = 10.473, *p* = 0.005), and freshly made beverages (*H* = 14.314, *p* = 0.01) is highest in remote areas, followed by urban central areas, and lowest in suburban areas.

### 3.3 Influencing factors in individual and interpersonal level of the consumption of sugar-sweetened beverages

#### 3.3.1 Individual level

Among seventh grade students in Beijing, those with knowledge of SSB have a higher prevalence of consumption compared to those without such knowledge (89.2% vs. 85.6%, *p* < 0.05). In terms of attitudes, the prevalence of health awareness among students is 58.0%. Among students without health awareness, the prevalence of SSB consumption is higher than that of those with health awareness (92.7% vs. 89.0%, *p* < 0.05) (Table 4).

**Table 4:** Chi-square test of sugar-sweetened beverages consumption [*n* (%)].

Factors	Survey sample (*n* = 1,212)	Consumption of sugar-sweetened beverages		*χ* ^2^	*P*-value
		Yes	No		
Individual level					
Knowledge of SSB				4.574	0.032
Yes	1,073(88.5)	979(91.2)	94(8.8)		
No	139(11.5)	119(85.6)	20(14.4)		
Health awareness				4.702	0.030
Yes	703(58.0)	626(89.0)	77(11.0)		
No	509(42.0)	472(92.7)	37(7.3)		
Self-efficacy				2.422	0.120
Yes	639(52.7)	571(89.4)	68(10.6)		
No	573(47.3)	527(92.0)	46(8.0)		
Positive attitude				2.149	0.143
Yes	687(56.7)	615(89.5)	72(20.5)		
No	525(43.3)	483(92.0)	42(8.0)		
Good dietary habits				0.672	0.413
Yes	679(56.0)	611(90.0)	68(10.0)		
No	533(44.0)	487(91.4)	46(8.6)		
Average daily water intake				0.501	0.779
<1,000 mL	217(17.9)	194(89.4)	23(10.6)		
1,000 ~ 1,500 mL	360(29.7)	326(90.6)	34(9.4)		
>1,500 mL	635(52.4)	578(91.0)	57(9.0)		
Average daily outdoor activity time				4.036	0.258
<30 min	112(9.2)	96(85.7)	16(14.3)		
30 min ~ 1 h	336(27.7)	303(90.2)	33(9.8)		
1 ~ 2 h	474(39.1)	435(91.8)	39(8.2)		
≥2 h	290(24.0)	264(91.0)	26(9.0)		
Interpersonal Level					
Peer consumption				8.675	0.003
Yes	1,146(94.6)	1,045(91.2)	101(8.8)		
No	66(5.4)	53(80.3)	13(19.7)		
Choosing to purchase SSB Due to peers’ purchases				19.476	<0.001
Yes	683(56.4)	641(93.9)	42(6.1)		
No	529(43.6)	457(86.4)	72(13.6)		
Sharing beverages				20.071	<0.001
Yes	858(70.8)	798(93.0)	60(7.0)		
No	354(29.2)	300(84.7)	54(15.3)		
Parents’ knowledge				0.296	0.586
Yes	1,131(93.3)	1,026(90.7)	105(9.3)		
No	81(9.4)	72(88.9)	9(11.1)		
Parents’ attitude				5.986	0.014
Support	557(46.0)	517(92.8)	40(7.2)		
Not support	655(54.0)	581(88.7)	74(11.3)		
Parents’ consumption				47.933	<0.001
Yes	1,082(89.3)	1,002(92.6)	80(7.4)		
No	130(10.7)	96(73.8)	34(26.2)		
Environmental Level					
Obtaining from supermarkets				0.095	0.758
Convenience	897(74.0)	814(90.7)	83(9.3)		
Inconvenient	315(26.0)	284(90.2)	31(9.8)		
Obtaining from convenience stores				0.000	0.990
Convenience	1,084(89.4)	982(90.6)	102(9.4)		
Inconvenient	128(10.6)	116(90.6)	12(9.4)		
Obtaining from vending machines				0.020	0.887
Convenience	688(56.7)	624(90.7)	64(9.3)		
Inconvenient	524(43.3)	474(90.5)	50(9.5)		
Obtaining from markets				0.208	0.648
Convenience	806(66.5)	728(90.3)	78(9.7)		
Inconvenient	406(33.5)	370(91.1)	36(8.9)		
Obtaining from restaurants				0.089	0.766
Convenience	976(80.5)	883(90.5)	93(9.5)		
Inconvenient	236(19.5)	215(91.1)	21(8.9)		
Points of sale for SSB within 100 m around campus				0.425	0.514
Yes	274(22.6)	251(91.6)	23(8.4)		
No	938(77.4)	847(90.3)	91(9.7)		
Policy Level					
Impact of health warning labels set up at point of sale on beverage purchases				23.984	<0.001
Buy a bit more	28(2.3)	23(82.1)^a,b^	5(17.9)^a,b^		
Do not change purchase plans	168(22.0)	248(92.5)^b^	20(7.5)^b^		
Buy a bit less	306(25.2)	280(91.5)^b^	26(8.5)^b^		
Choose low-sugar or sugar-free beverages	431(35.5)	401(93.0)^b^	30(7.0)^b^		
Give up purchasing	179(15.0)	146(81.6)^a^	33(18.4)^a^		
After seeing the health warning labels, discourage family or friends from drinking beverages				5.497	0.019
Yes	639(52.7)	567(88.7)	72(11.3)		
No	573(47.3)	531(92.7)	42(7.3)		
See health warning labels for SSB set up at points of sale				0.155	0.694
Yes	606(50.0)	551(90.9)	55(9.1)		
No	606(50.0)	547(90.3)	59(9.7)		
The significance of setting corresponding health warning labels for developing good dietary habits				1.985	0.159
Significant	846(69.8)	773(91.4)	73(8.6)		
Not significant	366(30.2)	325(88.8)	41(11.2)		

a, b indicated subsets of each variable after LSD pairwise comparison; different letters indicate statistically significant differences.

#### 3.3.2 Interpersonal level

##### 3.3.2.1 The influence of peers on students’ sugar-sweetened beverages consumption

94.6% of students have peers who engage in SSB consumption, and those whose peers consume SSB have a higher prevalence of SSB consumption than those whose peers do not (91.2% vs. 80.3%, *p* < 0.05). 56.4% of students choose to purchase SSB because their peers do, and those influenced by peers have a higher prevalence of SSB consumption than those not influenced (93.9% vs. 86.4%, *p* < 0.05). 70.8% of students share SSB with peers, and those who engage in sharing behavior have a higher prevalence of SSB consumption than those who do not (93.0% vs. 84.7%, *p* < 0.05) (Table 4).

##### 3.3.2.2 The influence of parents on students’ consumption of sugar-sweetened beverages

54% of parents do not support their children consuming SSB. Students whose parents support SSB consumption exhibit a higher prevalence of such consumption compared to those whose parents do not (92.8% vs. 88.7%, *p* < 0.05). The prevalence of SSB consumption among parents themselves is 89.3%. Students with parents who consume SSBs have a higher prevalence of SSB consumption than those whose parents do not consume them (92.6% vs. 73.8%, *p* < 0.05) (Table 4).

##### 3.3.2.3 The influence of teachers on students’ consumption of sugar-sweetened beverages

Qualitative interviews with teachers provided further insight into factors influencing students’ SSB consumption. Overall, teachers tended to express negative attitudes toward students’ consumption of SSB, emphasizing potential health risks, although a small proportion considered moderate intake acceptable. As one participant noted, “Sugar-sweetened beverages are harmful to health and may contribute to obesity and diabetes, so they are not recommended for students” (A12).

At the behavioral level, teachers reported that students’ consumption patterns were shaped by both individual habits and contextual factors. Some students were able to reduce intake or switch to alternatives such as low-sugar beverages or water, while seasonal variation was also observed, with higher consumption during warmer periods. For example, one teacher mentioned, “Some students choose low-sugar drinks or prefer drinking water instead” (A13).

Schools were identified as key settings for shaping students’ dietary behaviors. Teachers indicated that knowledge related to SSB consumption was mainly delivered through formal curricula and health promotion activities, including classroom teaching, lectures, and school-based campaigns. As one participant described, “Topics related to SSB and nutrition are covered in the curriculum” (A5).

In addition, participants highlighted the importance of broader intervention strategies, including strengthening family–school collaboration, improving health education, and implementing policy-level measures such as warning labels and restrictions on advertising. One teacher suggested, “Warning labels could be added to packaging to help students better understand the sugar content” (A9).

Qualitative findings emphasized the important roles of social norms, educational environments, and policy-related factors in shaping students’ snack and beverage consumption.

### 3.4 Environmental level

#### 3.4.1 Community food environment

In terms of the community food environment related to students’ consumption of SSB, 74.0% of students conveniently obtain SSB from large and medium-sized supermarkets, 89.4% from convenience stores, 56.7% from vending machines, 66.5% from farmers’ markets or vegetable markets, and 80.5% from restaurants (Table 4).

#### 3.4.2 Surrounding campus environment

22.6% of students reported the presence of SSB selling points within 100 m of their campus (Table 4).

### 3.5 Policy level

Interview results show that the schools involved in the survey strictly adhere to the relevant policies of Beijing and do not have SSB selling points on campus. In terms of implementing health education courses, the primary approach is through formal curriculum education. The curriculum covers a series of classes, including health education, moral and legal education, physical education, and biology. Schools regularly conduct health talks, set up bulletin boards for health education, and invite experts from relevant fields to give lectures on campus. Teachers report that students have a high level of acceptance and can understand and absorb the related knowledge well. In response to the demand for intervention measures regarding students’ consumption of SSB, most teachers mention the need to strengthen cooperation between home and school to jointly reduce students’ SSB consumption. It is important to cultivate parents’ awareness of health to serve as good role models for their children. Teachers also propose enhancing social practice activities, allowing students to visit beverage processing plants, and through field visits and hands-on experiments, experience the sugar content in SSB, thereby reducing their consumption. Furthermore, it is crucial to enhance social promotion, not only to strengthen health knowledge dissemination but also to increase awareness of the health risks posed by SSB, helping students recognize their dangers. Suggestions include increasing market regulation efforts, restricting advertising content and broadcast times, and establishing warning labels at selling locations and on packaging.

Quantitative survey results indicate that 75.7% of students exhibit positive behaviors due to health warning labels. Among them, 25.2% of students reduce their consumption of SSB because of health warning labels set up in sales venues, 35.5% choose other low-sugar drinks as substitutes, and 15.0% give up purchasing SSB. Additionally, 52.7% of students discourage those around them from consuming beverages after seeing the warnings. The rate of students who discourage others from consuming SSB is higher than that of students who do not, with the differences being statistically significant (*p* < 0.05) (Table 4).

### 3.6 Logistic regression analysis of students’ sugar-sweetened beverages consumption

The binary logistic regression model demonstrated adequate fit to the data, as indicated by Hosmer-Lemeshow test (*p* = 0.603). All variance inflation factors (VIFs) were below 5, suggesting that multicollinearity was not a concern. Logistic regression results indicate that students with knowledge of SSBs are 1.881 times more likely to consume these beverages than students without such knowledge (*OR* = 1.881, 95% *CI* [1.082–3.270]). Students who purchase SSB due to peer influence are 1.803 times more likely to consume them than those who do not (*OR* = 1.803, 95% *CI* [1.167–2.785]). Students who share drinks with peers are 1.841 times more likely to consume SSB than those who do not share (*OR* = 1.841, 95% *CI* [1.199–2.826]). Students whose parents support the consumption of SSBs are 1.629 times more likely to consume them than those whose parents do not support it (*OR* = 1.629, 95% *CI* [1.072–2.476]). Students whose parents themselves consume SSBs are 3.998 times more likely to consume them than those whose parents do not (*OR* = 3.998, 95% *CI* [2.500–6.395]) (Table 5).

**Table 5:** Logistic regression analysis of sugar-sweetened beverages consumption.

Factors	*β*	S. E.	Wald *χ*^2^	*P*	OR	95%CI
Knowledge of SSB						
Yes	0.632	0.282	5.016	0.025	1.881	1.082–3.270
No (Reference)						
Choosing to purchase SSB due to peers’ purchases						
Yes	0.589	0.222	7.050	0.008	1.803	1.167–2.785
No (Reference)						
Sharing beverages						
Yes	0.610	0.219	7.782	0.005	1.841	1.199–2.826
No (Reference)						
Parents’ attitude						
Support	0.488	0.214	5.222	0.022	1.629	1.072–2.476
Not support (Reference)						
Parents’ consumption						
Yes	1.386	0.240	33.443	<0.001	3.998	2.500–6.395
No (Reference)						
Constant	0.012	0.357	0.001	0.973	1.012	

OR, odds ratio; CI, confidence interval. The outcome variable was SSB consumption (1 = consumed any SSB in the past week, 0 = no consumption).

Using the frequency of SSB consumption as the outcome variable to capture high-risk intake, we conducted a binary logistic regression analysis to identify factors associated with high-frequency consumption. The binary logistic regression model demonstrated adequate fit to the data, as indicated by Hosmer-Lemeshow test (*p* = 0.115). All variance inflation factors (VIFs) were below 5, suggesting that multicollinearity was not a concern. In the final multivariable logistic regression model, several interpersonal and individual factors were significantly associated with high-frequency SSB consumption. Students who reported purchasing SSBs in response to peers were more likely to be high-frequency consumers (*OR* = 1.415, 95% *CI*: 1.100–1.820, *p* = 0.007), and those who shared beverages with classmates also had a higher likelihood of frequent consumption (*OR* = 1.813, 95% *CI:* 1.386–2.370, *p* < 0.001). Parental SSB consumption was positively associated with adolescents’ consumption, with students whose parents consumed SSBs more likely to be high-frequency consumers (*OR* = 1.659, 95% *CI*: 1.140–2.414, *p* = 0.008). In contrast, students with health-oriented attitudes (perceiving them as harmful) toward SSBs were less likely to be high-frequency consumers (*OR* = 0.681, 95% *CI*: 0.534–0.870, *p* = 0.002) (Supplementary Table S3).

## 4 Discussion

The present study identified a high prevalence of sugar-sweetened beverage consumption among seventh grade students in Beijing, with 90.6% reporting consumption in the past week and 61.5% consuming SSBs on a daily basis. These findings are broadly consistent with earlier multi-province surveys conducted in China (22), but the level of daily consumption observed in this study is substantially higher than that reported in Shanghai and national dietary surveys (23, 24). In addition, the estimated daily intake in this population was 392.86 (178.57, 809.28) mL/day, exceeding national averages for adolescents (23), suggesting that SSB consumption among students in Beijing is not only widespread but also characterized by frequent and habitual intake. In recent years, the rapid expansion of freshly prepared beverages (e.g., milk tea and fruit tea) has increased adolescents’ exposure to SSBs, which may have contributed to the higher consumption levels observed in this study. Regional differences were also observed, with higher consumption in central urban and remote urban areas compared with suburban areas, in line with previous findings (25). Taken together, these results indicate that SSB consumption remains a prominent issue among adolescents and may reflect sustained consumption patterns rather than occasional intake.

With regard to individual-level factors, this study found a positive association between SSB-related knowledge and consumption, which differs from previous studies reporting either no association or a protective effect of knowledge (26). Although the overall level of SSB-related knowledge in this study was relatively high (88.5%), exceeding that reported among students in Shenzhen and among the general population in China (27, 28), this did not correspond to lower levels of consumption. This finding suggests a potential disconnect between knowledge and behavior. Reverse causality cannot be ruled out, as students with higher levels of consumption may have greater exposure to SSB-related information. Besides, the knowledge measure used in this study may primarily capture awareness or exposure to information rather than the depth of understanding or the ability to translate knowledge into behavior change. This finding highlights a potential gap between knowledge and behavior, suggesting that knowledge alone may be insufficient to influence consumption patterns.

At the environmental level, no significant association was identified between the measured food environment and SSB consumption, which differs from previous research (29, 30). This discrepancy may be partly attributable to differences in measurement approaches. In addition, in highly urbanized settings such as Beijing, the widespread availability of SSBs across different areas may reduce observable variation in environmental exposure, thereby attenuating measurable associations. Further research using more refined and context-specific indicators is needed to better capture environmental influences on consumption.

At the policy level, health warning labels on SSB products were identified as a potentially important strategy for reducing consumption. Strengthening the implementation of such policies at points of sale may contribute to reducing consumption by increasing awareness of health risks. Moreover, these measures may have broader effects by shaping social perceptions and norms related to SSB consumption. Qualitative findings from this study further highlighted the importance of school-based health education, the availability of standardized intervention materials, and collaboration among schools, families, and society. These findings suggest that institutional and policy support may play an important role in shaping the broader context in which individual and social behaviors occur.

In addition, the determinants of high-risk SSB consumption may differ from those of any consumption. Analyses using high-frequency consumption as an alternative indicator of potentially risky intake showed generally consistent patterns, suggesting that the observed associations were robust across different definitions of SSB consumption.

Overall, the findings of this study suggest that SSB consumption among adolescents is shaped by multiple factors operating across different levels. While knowledge was associated with consumption, interpersonal influences, particularly those related to peers and family, demonstrated stronger and more direct relationships with behavior. Environmental and policy factors may shape the broader context but are more difficult to capture using general indicators. These findings indicate that interventions focusing solely on improving knowledge may be insufficient, and that more comprehensive strategies addressing social influences, strengthening family and school engagement, and enhancing policy-level support may be more effective in reducing SSB consumption among adolescents.

## 5 Conclusion

The consumption rate of SSB among seventh grade students in Beijing reaches 90.6%, which is higher than the national level (68.3%) and previous research results in Beijing (43.6 to 67.3%). There are regional differences, with students in both central urban areas and remote urban areas having higher consumption rates than those in suburban areas.

Research results based on the social ecological model indicate that, at the individual level, there is a correlation between students’ consumption of SSB and their knowledge related to SSB. At the interpersonal level, purchasing SSB due to peer influence, sharing SSB among peers, parental support for students’ SSB consumption, and parents’ own consumption of SSB are all associated with higher consumption among students. At the environmental level, students can conveniently obtain beverages from community convenience stores and restaurants. At the policy level, setting health warning labels at points of sale for SSB influences students’ consumption.

In the future, strengthening policies to establish health warning labels at sales locations for SSB, creating student-centered collaborative health promotion policies involving schools and families, and implementing multi-dimensional intervention strategies centered around students, parents, and teachers can reduce students’ consumption of SSB and enhance their physical health.

## 6 Limitations

This study has several limitations. First, the cross-sectional design precludes any inference of causal relationships between identified factors and SSB consumption. Second, both SSB intake and consumption frequency were based on self-reported data, which may be subject to recall bias. Finally, although multiple variables were adjusted for in the regression models, residual confounding from unmeasured factors, such as household dietary environment and individual preferences, cannot be excluded.

## Data Availability

The datasets are not publicly available due to restrictions related to participant privacy and ethical approval, but are available from the corresponding author on reasonable request.
