# Supplementary material for: Research on the consumption of sugar-sweetened beverages among seventh grade students in Beijing based on the social ecological model
Source: Front Nutr. 2026 Jun 23;13:1817462. doi: 10.3389/fnut.2026.1817462 (PMC13337499; doi:10.3389/fnut.2026.1817462)
Supplement: Supplementary file 1 [file Table_1.DOCX]

# **Additional files 1. Interview Guide (Chinese and English)**

**Additional files 1A. Focus Group Interview Guide**

**EN1. What are the potential health risks of sugar-sweetened beverage (SSB) consumption among students, based on your knowledge?**
CH1. 您知道的含糖饮料消费对学生有哪些危害？

**EN2. Do you think students’ consumption of SSBs should be controlled? If so, to what level?**
CH2. 您认为是否应该控制学生含糖饮料的消费？如果控制，应该控制在什么水平？

**EN3. What is students’ level of awareness regarding SSBs? Do they understand the potential health risks? Are they aware that SSBs may lead to obesity, diabetes, or other health problems?**
CH3. 学生对含糖饮料的认知程度如何？他们了解含糖饮料对健康的潜在危害吗？是否有学生明确意识到含糖饮料可能引发肥胖、糖尿病等健康问题？

**EN4. What is your attitude toward students’ consumption of SSBs?**
CH4. 您如何看待学生含糖饮料消费的行为，对此持有什么态度？

**EN5. In your interactions with students, have you observed SSB consumption?**
CH5. 您在与学生的接触中，是否看到过学生消费含糖饮料？

**EN6. What are students’ attitudes toward SSBs? Are they willing to reduce or avoid consumption? Are they open to healthier alternatives or increasing water intake?**
CH6. 学生对于含糖饮料的态度是怎样的？他们是否愿意主动减少或避免饮用含糖饮料？是否有学生愿意尝试替代性健康饮料或者增加水的摄入量？

**EN7. What is the current status of health education on SSBs in schools? (Content, format, frequency, etc.)**
CH7. 关于含糖饮料的健康课程在学校的开展情况如何？（涉及内容、形式及频率等）

**EN8. How do students respond to these health courses? Are there indications of their understanding and acceptance?**
CH8. 学生对这些健康课程的反馈如何？是否有反馈表明学生对课程内容的接受度或理解度？

**EN9. What interventions do you think could effectively reduce students’ SSB consumption?**
CH9. 根据目前情况，您认为有哪些有效的干预措施可以帮助学生减少含糖饮料的消费？

**EN10. Are there any specific programs or activities aimed at promoting healthy dietary behaviors among students?**
CH10. 是否有针对学生的具体计划或活动，以促进健康饮食习惯的形成？

**EN11. What resources and support do teachers (e.g., head teachers, health educators) need? Does the school or other institutions provide such support (e.g., teaching materials, training)?**
CH11. 班主任和健康老师在这方面需要哪些支持和资源？学校或其他机构是否提供相应支持（如教材、培训等）？

**EN12. What are your future plans or expectations for improving students’ dietary behaviors and health awareness?**
CH12. 对于未来，您有哪些计划或期望，以进一步改善学生的饮食习惯和健康意识？

## EN13.Basic Information

## CH13. 基本信息

- **Participant role:** School leader / Head teacher / Health teacher / Logistics staff / Other
  访谈对象：校领导 / 班主任 / 健康老师 / 后勤老师 / 其他
- **Gender:** Male / Female
  性别：男 / 女
- **Age (years):** ______
  年龄：______
- **Education level:** ______
  学历：______
- **Years of employment:** ______
  就职年限：______

**Additional files 1B. In-depth Interview Guide**

**EN1. Are there any data or surveys on students’ SSB consumption within the school?**
CN1. 是否有数据或调查显示学生在校园内的含糖饮料消费情况？

**EN2. What is students’ level of awareness regarding SSBs? Do they understand the potential health risks?**
CN2. 学生对含糖饮料的认知程度如何？他们是否了解其健康危害？

**EN3. What is your attitude toward students’ SSB consumption?**
CN3. 您如何看待学生含糖饮料消费的行为？

**EN4. Are there any school policies or regulations regarding SSB consumption?**
CN4. 在学校环境中，是否存在饮用含糖饮料的行为规范或限制？

**EN5. What are students’ attitudes? Are they willing to reduce consumption or adopt healthier alternatives?**
CN5. 学生是否愿意减少含糖饮料摄入或选择健康替代品？

**EN6. Are there school-based health education programs on SSBs? How are they designed and implemented?**
CN6. 学校是否有针对含糖饮料的健康教育课程？如何设计与开展？

**EN7. Is there a systematic health education plan addressing SSB risks and promoting healthier alternatives?**
CN7. 是否有系统性的健康教育计划？

**EN8. Based on students’ behaviors and attitudes, what interventions do you consider effective?**
CN8. 根据学生情况，您认为哪些干预措施可能有效？

**EN9. Should the frequency or content of health education be enhanced?**
CN9. 是否需要增加健康教育频率或内容？

**EN10. Does the school need additional resources (e.g., materials, trainers, campaigns)?**
CN10. 学校是否需要更多资源支持？

**EN11. Are there external partners that could support health education?**
CN11. 是否有外部合作资源？

**EN12. What are the future plans for improving students’ health behaviors?**
CN12. 未来是否有进一步策略改善学生健康行为？

## EN13.Basic Information

## CH13. 基本信息

- **Participant role:** School leader / Head teacher / Health teacher / Logistics staff / Other
  访谈对象：校领导 / 班主任 / 健康老师 / 后勤老师 / 其他
- **Gender:** Male / Female
  性别：男 / 女
- **Age (years):** ______
  年龄：______
- **Education level:** ______
  学历：______
- **Years of employment:** ______
  就职年限：______
