# Supplementary material for: Research on the consumption of sugar-sweetened beverages among seventh grade students in Beijing based on the social ecological model
Source: Front Nutr. 2026 Jun 23;13:1817462. doi: 10.3389/fnut.2026.1817462 (PMC13337499; doi:10.3389/fnut.2026.1817462)
Supplement: Supplementary file 2 [file Table_2.DOCX]

Supplementary Material

# Supplementary Tables

**Supplementary Table 1. Definition and coding of study variables**

| Category | Variable | Measurement | Coding | Example |
| --- | --- | --- | --- | --- |
| Outcome | SSB consumption (primary outcome) | Consumption in past week | 0 = No, 1 = Yes | Have you consumed sugar-sweetened beverages within the past week? |
|  | High-frequency SSB consumption (secondary outcome) | Frequency of SSB consumption during the past week | 0 = < 7 times/week, 1 = ≥ 7 times/week | Over the past week, how many times have you consumed sugar-sweetened beverages? |
| SSB consumption (descriptive) | SSB frequency | Self-reported frequency (standardized to weekly) | Continuous (times/day) | Over the past week, which types of sugar-sweetened beverages did you consume, and what was the consumption frequency for each type? |
|  | Daily intake | Estimated from frequency × amount (mL) | Continuous (mL/day) | Over the past week, which types of sugar-sweetened beverages did you consume, on average, how many sugary drinks did you consume each time? |
| Individual | Knowledge (student) | 10 items, 1 point each | 0–10;  ≥8 = adequate | Does carbonated beverages affect the bone development of children? |
|  | Attitudes (student) | 17 Likert items across 4 domains | 0–4 per item; dichotomized by median | I think drinking sugar-sweetened beverages is harmful to health. |
|  | Health behaviors | Water intake, outdoor activity | Continuous / categorical | The average amount of mineral water consumed in the past week. |
| Interpersonal | Peer influence | Peer consumption, purchase influence, sharing | Yes/No or ordinal | Would you buy a drink because your classmates are buying one? |
|  | Parental knowledge | 5 items, 1 point each | 0–5;  ≥4 = adequate | Sugary drinks fall under the category of high-sugar foods? |
|  | Parental attitudes | Likert scale items | 0–4 per item; dichotomized by median | I restrict my children from drinking sugary drinks. |
|  | Parental consumption | Past-week SSB consumption | 0 = No, 1 = Yes | Have you consumed sugar-sweetened beverages within the past week? |
| Environmental | Availability (residence) | SSB availability within 500 m | 0 = No, 1 = Yes | Is there any place selling sugary drinks within a 500-meter walking distance from your home? |
|  | Availability (school) | SSB sales within 100 m | 0 = No, 1 = Yes | On-site investigation |
| Policy | Warning label exposure | Exposure to SSB labels | 0 = No, 1 = Yes | Have you ever seen health warning signs in places where sugary drinks are sold? |
|  | Label influence | Perceived impact on purchasing | Ordinal | If you see the health warning sign, what would be your choice when purchasing sugary drinks? |

**Supplementary Table 2. Consumption of Different Types of sugar-sweetened beverages [n (%)]**

| **Pre-packaged Beverages** | **Survey Sample （n=1212）** | **Freshly made Beverages** | **Survey Sample （n=1212）** |
| --- | --- | --- | --- |
| 100% fruit and vegetable juice | 580（47.9） | coffee | 257（21.3） |
| non-100% fruit and vegetable juice | 476（39.3） | fruit tea | 509（42.0） |
| carbonated beverage | 521（43.0） | pure tea | 497（41.1） |
| tea drink | 586（48.4） | milk tea | 474（39.2） |
| dairy beverage | 395（32.6） | fruit juice | 448（37.0） |
| plant-protein drink | 272（22.5） | specialty tea | 235（19.4） |
| special use beverage | 453（37.4） | yogurt/milk | 353（29.2） |
| coffee | 198（16.4） | carbonated drink | 290（24.0） |
| other pre-packaged beverage | 296（12.2） | other freshly made beverage | 38（3.2） |
| **Total** | 1022（84.4） | **Total** | 964（79.6） |

**Supplementary Table 3. Logistic regression analysis of factors associated with high-frequency SSB consumption**

| **Factors** | **β** | **S.E.** | **Waldχ2** | ***P*** | ***OR*** | **95%*CI*** |
| --- | --- | --- | --- | --- | --- | --- |
| **Health Awareness** |  |  |  |  |  |  |
| Yes | -0.384 | 0.125 | 9.467 | 0.002 | 0.681 | 0.534-0.870 |
| No (Reference) |  |  |  |  |  |  |
| **Choosing to Purchase sugar-sweetened beverages Due to Peers’ Purchases** |  |  |  |  |  |  |
| Yes | 0.347 | 0.128 | 7.317 | 0.007 | 1.415 | 1.100-1.820 |
| No (Reference) |  |  |  |  |  |  |
| **Sharing Beverages** |  |  |  |  |  |  |
| Yes | 0.595 | 0.137 | 18.877 | ＜0.001 | 1.813 | 1.386-2.370 |
| No (Reference) |  |  |  |  |  |  |
| **Parents’ Consumption** |  |  |  |  |  |  |
| Yes | 0.506 | 0.191 | 6.987 | 0.008 | 1.659 | 1.140-2.414 |
| No (Reference) |  |  |  |  |  |  |
| **Constant** | -0.358 | 0.215 | 2.766 | 0.096 | 0.699 |  |

Note: OR = odds ratio; CI = confidence interval ; The outcome variable was high-frequency SSB consumption (1 = high frequency, 0 = low frequency)
